# Supplementary material for: Regulation of centrosome size by the cell-cycle oscillator in Drosophila embryos
Source: EMBO J. 2024 Jan 17;43(3):5. doi: 10.1038/s44318-023-00022-z (PMC10898259; doi:10.1038/s44318-023-00022-z)
Supplement: Supplementary file 1 — Appendix with Supplementary Figures S1-S5 [file 44318_2023_22_MOESM1_ESM.pdf]

Appendix

Table of Contents:

Appendix Figure S1
Appendix Figure S2
Appendix Figure S3
Appendix Figure S4
Appendix Figure S5

Figure S1

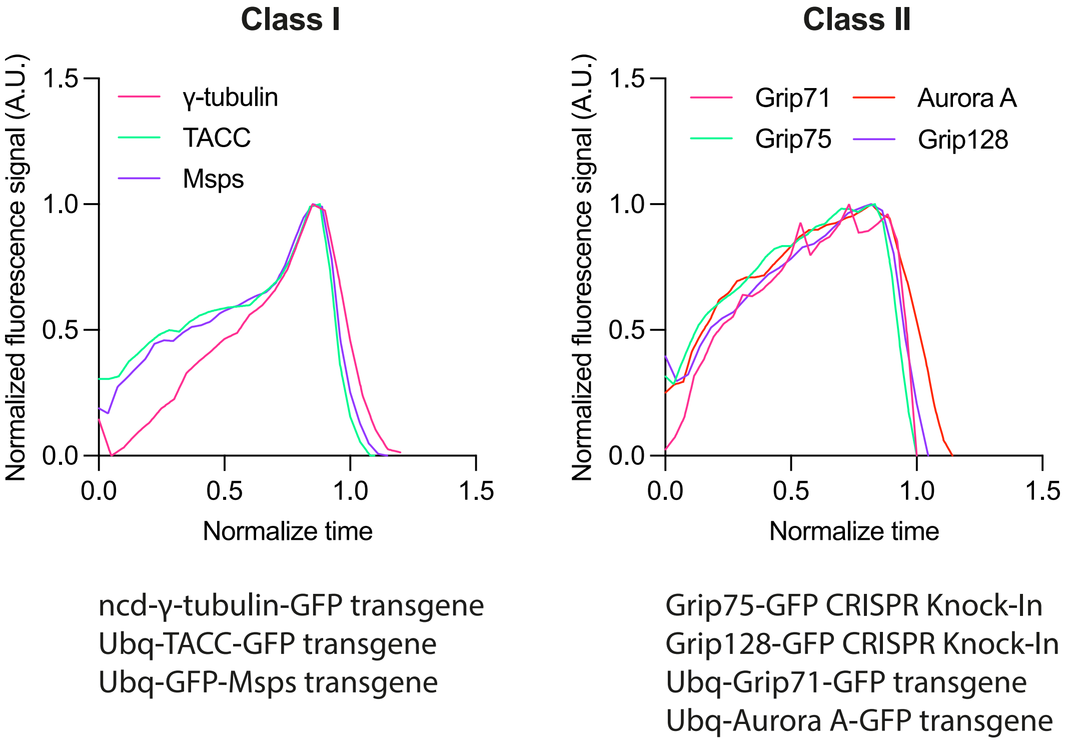

Comparison of Class I and Class II PCM-client growth kinetics during NC13. Graphs show the normalised mean centrosomal fluorescence intensity profiles of the Class I and Class II PCM-client proteins during NC13. The data (taken from Figure 1) has been normalised so that both the minimum and maximum centrosome-fluorescence-intensity, and the time from centrosome separation at the start of S-phase to NEB at the end of S-phase, have been set to 0 and 1, respectively, for each client protein. From this analysis it can be seen that all the proteins in each Class exhibit very similar dynamics, even though their expression in the embryo is driven using a variety of either endogenous or heterogeneous promotors: Grip75-GFP and Grip128-GFP are CRISPR knock-ins at the endogenous loci; TACC-GFP, GFP-Msps, Grip71-GFP and Aurora A-GFP are all transgenes driven from the ubiquitin promotor (Ubq), while γ-tubulin-GFP is a transgene driven by the heterologous ncd-kinesin promoter.

Figure S2

A Spd-2

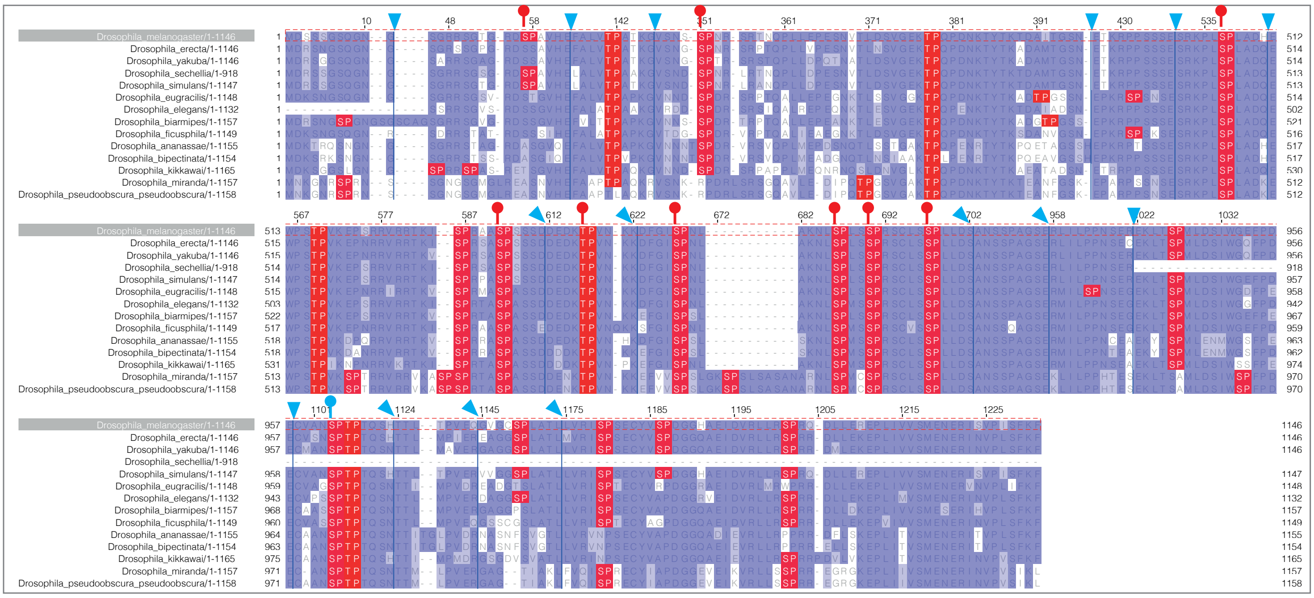

B Cnn

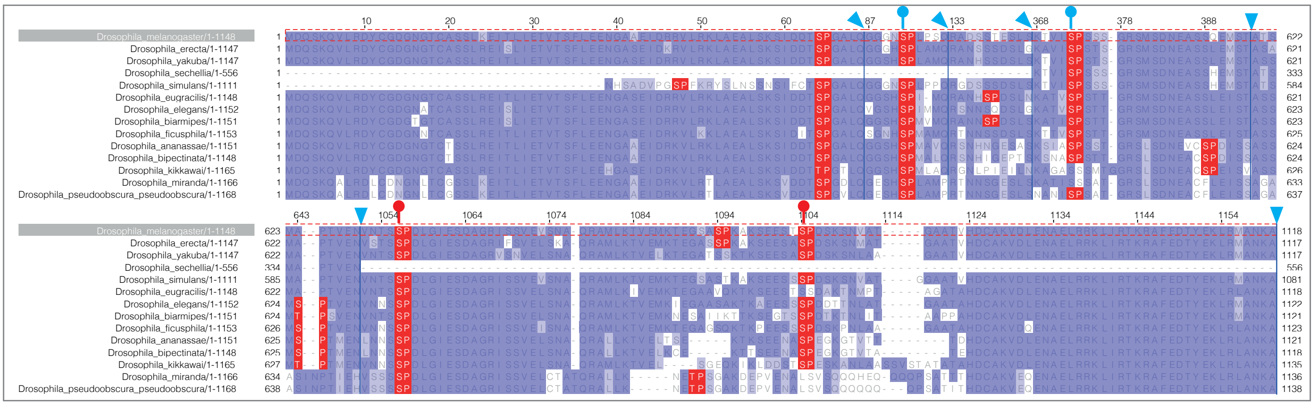

Analysis of potential Cdk/Cyclin phosphorylation sites (S/T-P motifs) in Spd-2 and Cnn. Panels show multiple sequence alignments (MSA) of Spd-2 **(A)** and Cnn **(B)**, highlighting potential S/T-P motifs (*red*) present in the D. melanogaster proteins (top row of MSAs) and in 13 other Drosophila species. The purple shading represents a BLOSUM62 conservation score (darker shading indicating higher conservation). Coloured knobs indicate that the S/T-P site has been found to be phosphorylated in Mass Spectroscopy screens in embryos of D. melanogaster (*red*) or in another Drosophila species (*blue*). The blue arrows above the MSA indicate the boundaries of regions of the MSA that are not shown here as they do not contain any S/T-P motifs that were mutated in this study. The sequences of the different Drosophila species were aligned using MUSCLE with default parameters and visualised using Jalview 2.11.

Figure S3

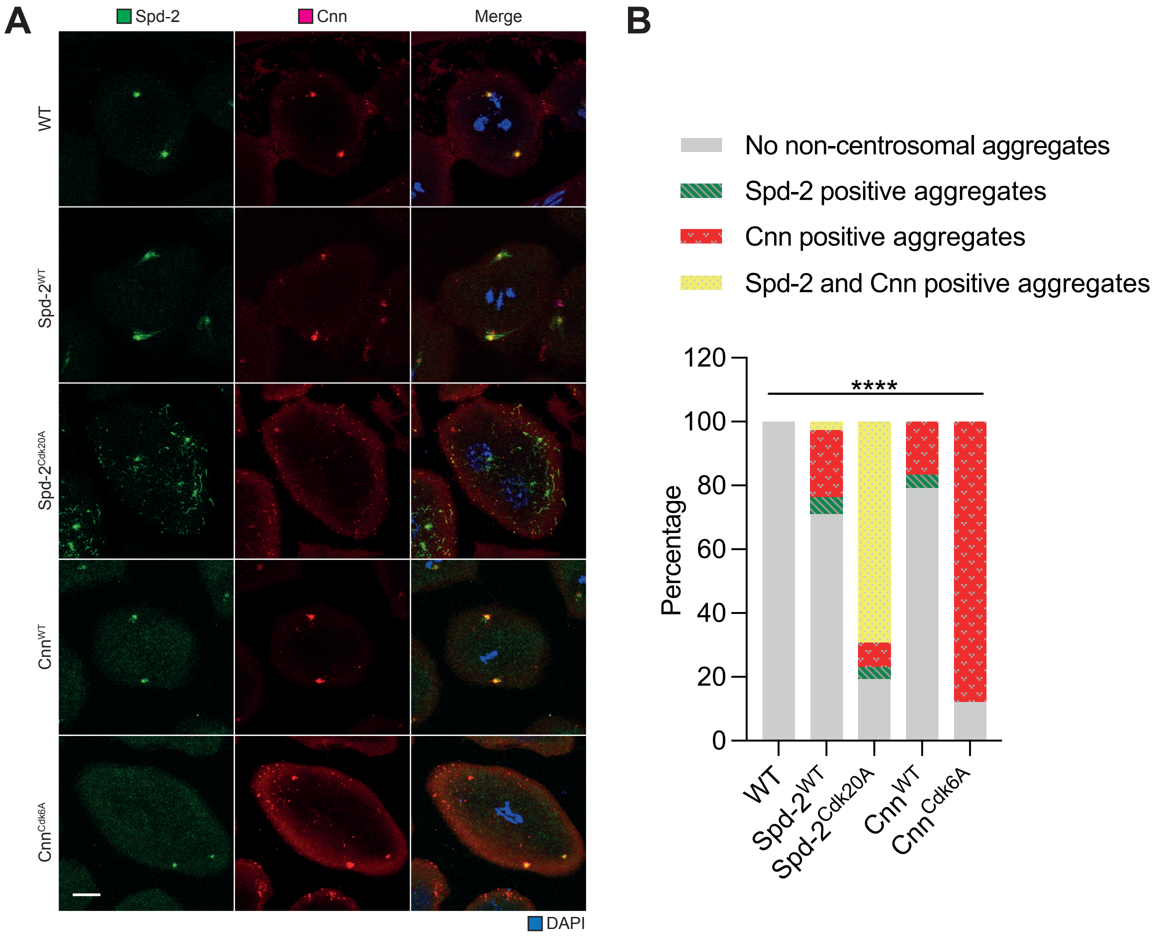

Analysis of meiosis in spermatocytes expressing Spd-2-Cdk20A-NG or NG-Cnn-Cdk6A. (A) Images show examples of spermatocytes in meiosis I stained to reveal the distribution of Spd-2 (*green*), Cnn (*red*) or DNA (*blue*) from WT males, or males transgenically expressing either WT Spd-2, Spd-2-Cdk20A, WT Cnn or Cnn-Cdk6A. The spermatocytes expressing Spd-2-Cdk20A or Cnn-Cdk6A often contained prominent cytoplasmic aggregates; the Spd-2-Cdk20A aggregates often also recruit Cnn, but the Cnn aggregates do not detectably recruit Spd-2—in agreement with previous data showing that Spd-2 recruits Cnn to the mitotic PCM, but Cnn does not recruit Spd-2 (Conduit et al, 2014b). Note also that the transgenic expression of WT Spd-2 seems to lead to the recruitment of extra Spd-2 to the centrioles. Scale bar=10 μm. **(B)** Bar chart quantifies the presence of these cytoplasmic aggregates in spermatocytes of the different genotypes—all data was scored blind. N=10-50 spermatocytes per genotype. The statistical significance of the proportion change was calculated using the original number of spermatocytes by Chi-square test (****: $P<0.0001$).

Figure S4

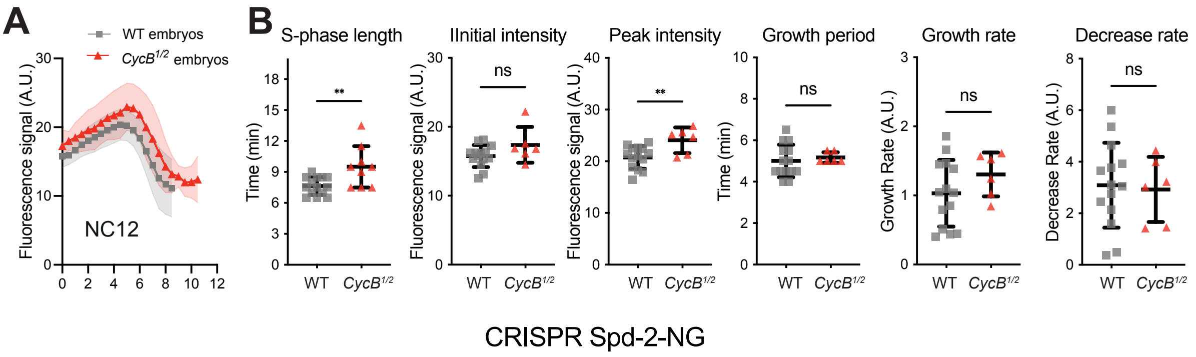

Further analysis of centrosomal Spd-2 growth kinetics in *CycB$^{1/2}$* embryos.
(A) Graph compares how the centrosomal fluorescence intensity (mean±SD) of WT Spd-2-NG changes over time in either *WT* or *CycB$^{1/2}$* embryos. **(B)** Scatter plots show the mean (±SD) of various cell cycle and centrosome-growth parameters derived from the data shown in (A). This experiment is essentially a repeat of the analysis shown in Figure 5E,F, except that the Spd-2-NG line analysed is a CRISPR/Cas9 knock-in of Spd-2-NG into the endogenous Spd-2 locus (a Ubq-Spd-2-NG transgene was used in the original analysis). This experiment was performed to illustrate how the difference in Spd-2-NG behaviour in *WT* and *CycB$^{1/2}$* embryos, although subtle, is reproducible; the broad shape of the curves and the trends in all of the parameters measured are the same in both experiments—even though most of these differences are not statistically significant. N=6-12 embryos and a total of n=~400-1000 centrosomes were analysed for each condition. Statistical comparisons were performed as described in the legend to Figure 3.

Figure S5

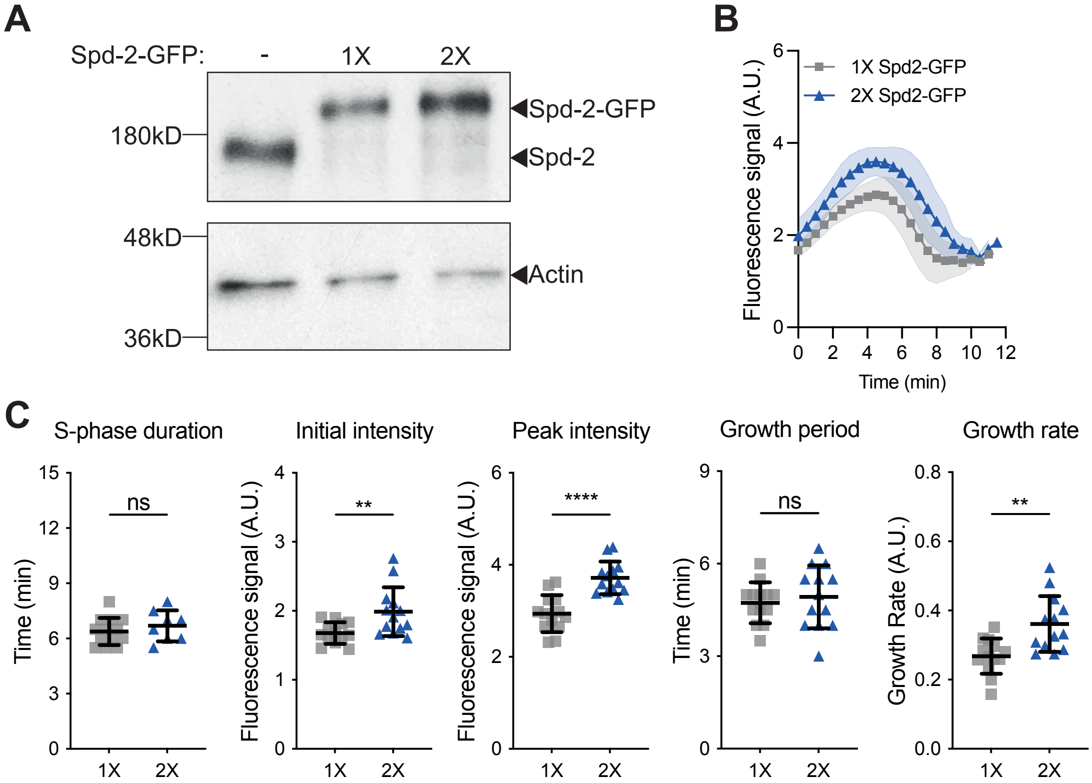

Changing the cytoplasmic concentration of Spd-2 changes the amount of Spd-2 recruited to centrosomes.

(A) Western blot shows Spd-2 and Spd-2-GFP protein-levels in either *WT* embryos, or in embryos laid by *Spd-2*$^{-/-}$ mutant females expressing 1 (1X) or 2 (2X) copies of a Ubq-Spd-2-GFP transgene. Actin serves as a loading control. **(B)** Graph shows how the centrosomal fluorescence intensity (mean±SD) of Spd-2-GFP changes over time during NC12 in embryos laid by *Spd-2*$^{-/-}$ mutant females expressing either 1 (1X) or 2 (2X) copies of a Ubq-Spd-2-GFP transgene. N=12-15 embryos and a total of n=~600-800 centrosomes were analysed for each condition. **(C)** Scatter plots compare various cell cycle and centrosome growth parameters derived from the data shown in (B). Statistical significance was assessed using an unpaired t-test (**: P<0.01, ***: P<0.001, ****: P<0.0001, ns: not significant).
