# Supplementary material for: Regulation of centrosome size by the cell-cycle oscillator in Drosophila embryos
Source: EMBO J. 2024 Jan 17;43(3):5. doi: 10.1038/s44318-023-00022-z (PMC10898259; doi:10.1038/s44318-023-00022-z)
Supplement: Supplementary file 3 — Expanded View Figures [file 44318_2023_22_MOESM3_ESM.pdf]

# Expanded View Figures

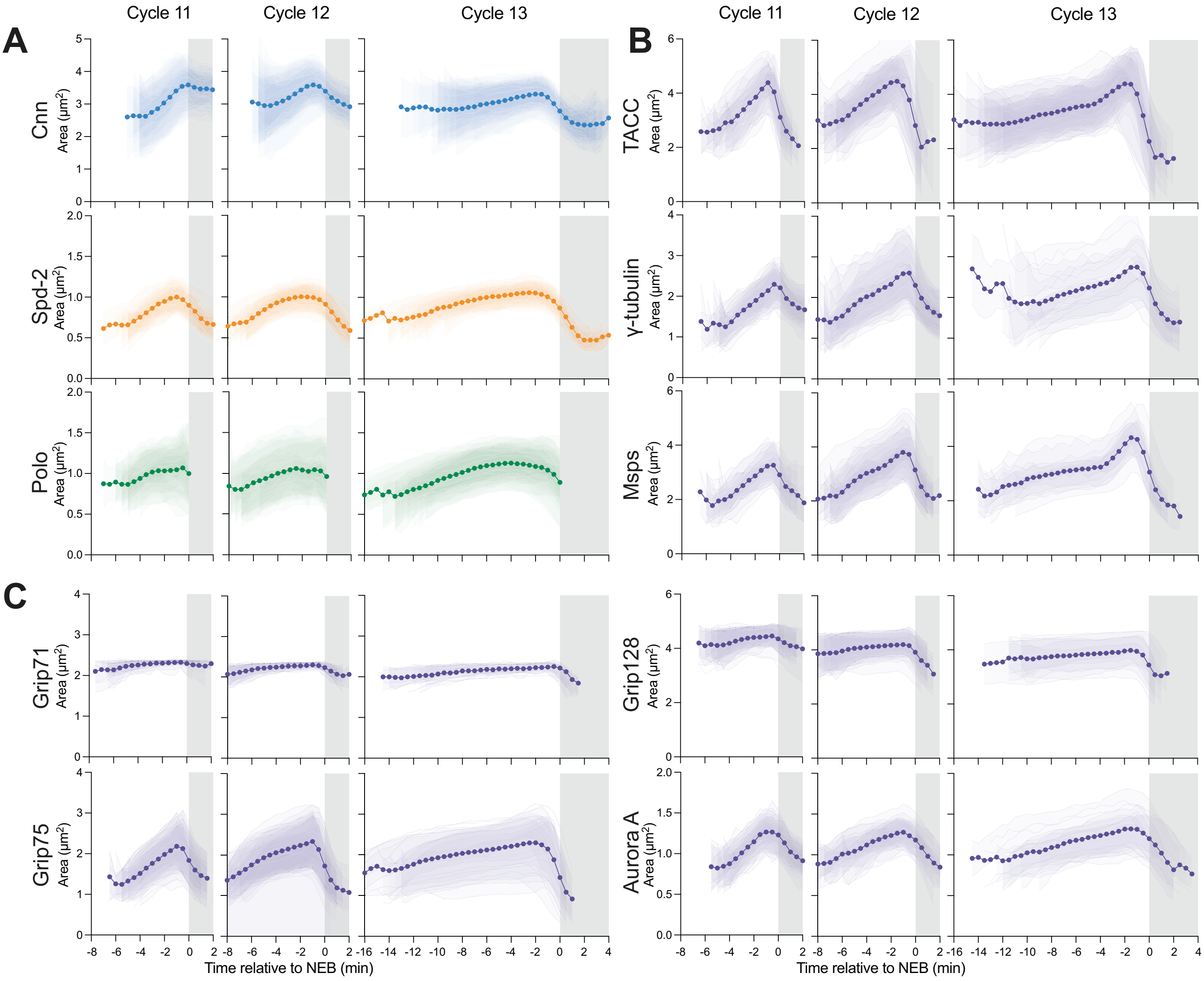


**Figure EV1. Analysis of centrosome growth kinetics measured by changes in centrosomal area during NC11, 12 and 13.**

(**A**) Graphs show how the mean centrosomal area (±SD of the data in each individual embryo shown in reduced opacity) of the PCM-scaffolding proteins Cnn, Spd-2 and Polo, varies during NC11, 12, and 13. These graphs were derived from the same embryos analysed in Fig. 1A. All individual embryo tracks were aligned to NEB ($t = 0$). The white parts of the graphs represent S-phase, and the grey parts represent mitosis. (**B**, **C**) Graphs show the same as (**A**) for the Class I (**B**) and Class II (**C**) PCM-client proteins (graphs derived from the same embryos analysed in Fig. 1B,C). Note that the Grip71- and Grip128-fluorescent fusion proteins were very dim. As a result, although their centrosomal distribution appeared very similar to Grip75 and γ-tubulin (Fig. 1A), our computational thresholding pipeline assigned them a larger area than these other proteins. This meant that the computationally calculated area of both proteins did not change very much during each nuclear cycle. $N = 7–15$ embryos analysed at each nuclear cycle for each marker with a total of $n = $ ~200–400, ~400–800, or ~600–1200 total centrosomes analysed at NC11, 12 and 13, respectively.

© The Author(s)

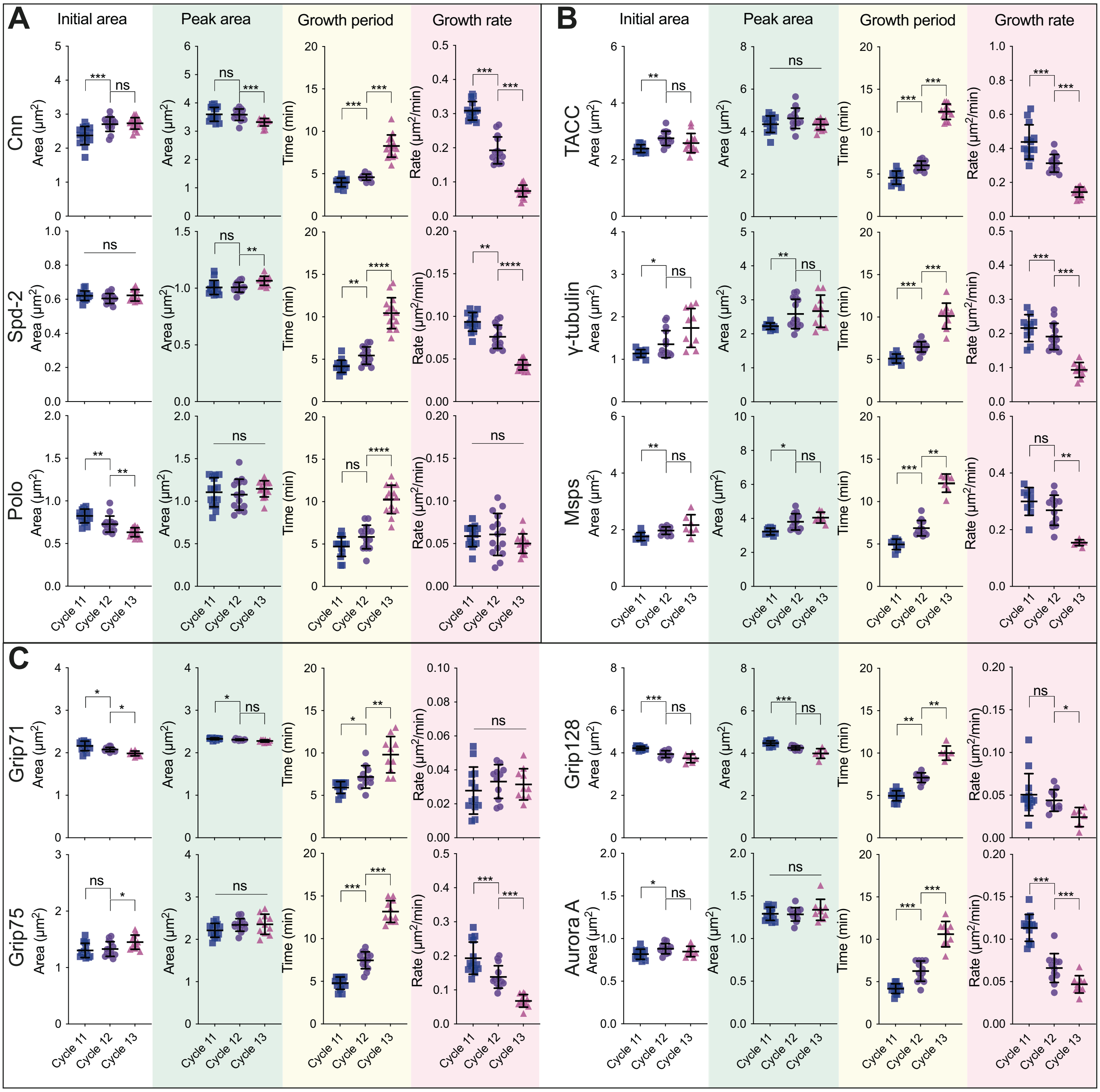


**Figure EV2. Analysis of centrosome growth parameters, measured by centrosome area, during NC11, 12 and 13.**

(**A**) Scatter plots show the mean (±SD) initial fluorescent intensity (left graphs), peak fluorescent intensity (boxed in green), growth period (boxed in yellow), and growth rate (boxed in pink) in NC11, 12, and 13 for the PCM-scaffolding proteins. (**B**, **C**) Scatter plots show the same as in (**A**) for the Class I (**B**) and Class II (**C**) PCM-client proteins. All plots were derived from the same embryos analysed in Fig. 1. Statistical comparisons were performed as described in the legend to Fig. 3.

 © The Author(s)

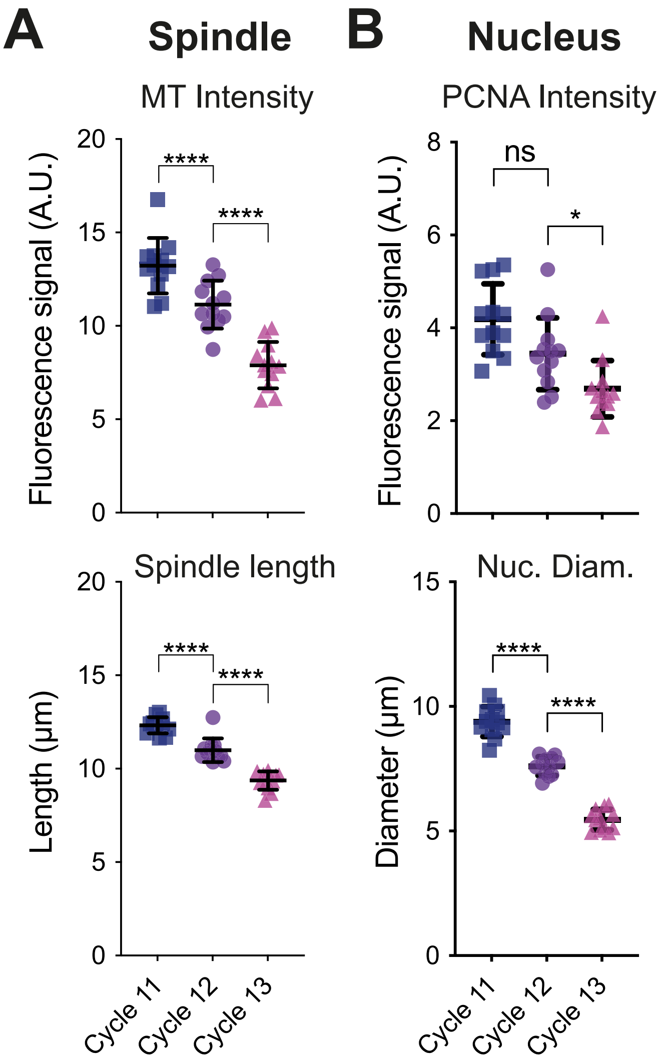


**Figure EV3. Comparison of how maximum spindle size and maximum nuclear size change during NC11, 12 and 13.**

(**A**) Scatter plots compare how the mean (±SD) fluorescence intensity of the metaphase mitotic spindle (top) and the mean (±SD) metaphase spindle length (bottom) vary during NC11, 12 and 13. (**B**) Scatter plots compare how the mean (±SD) fluorescence intensity of nuclear PCNA-RFP (top) and the mean (±SD) nuclear diameter (bottom) vary during NC11, 12 and 13. $N = 8$–12 embryos were analysed at each nuclear cycle with a total of $n = $ ~200–300, ~400–500, or ~600–800 total spindles/nuclei analysed at NC11, 12 and 13, respectively. Statistical comparisons were performed as described in the legend to Fig. 3.

© The Author(s)

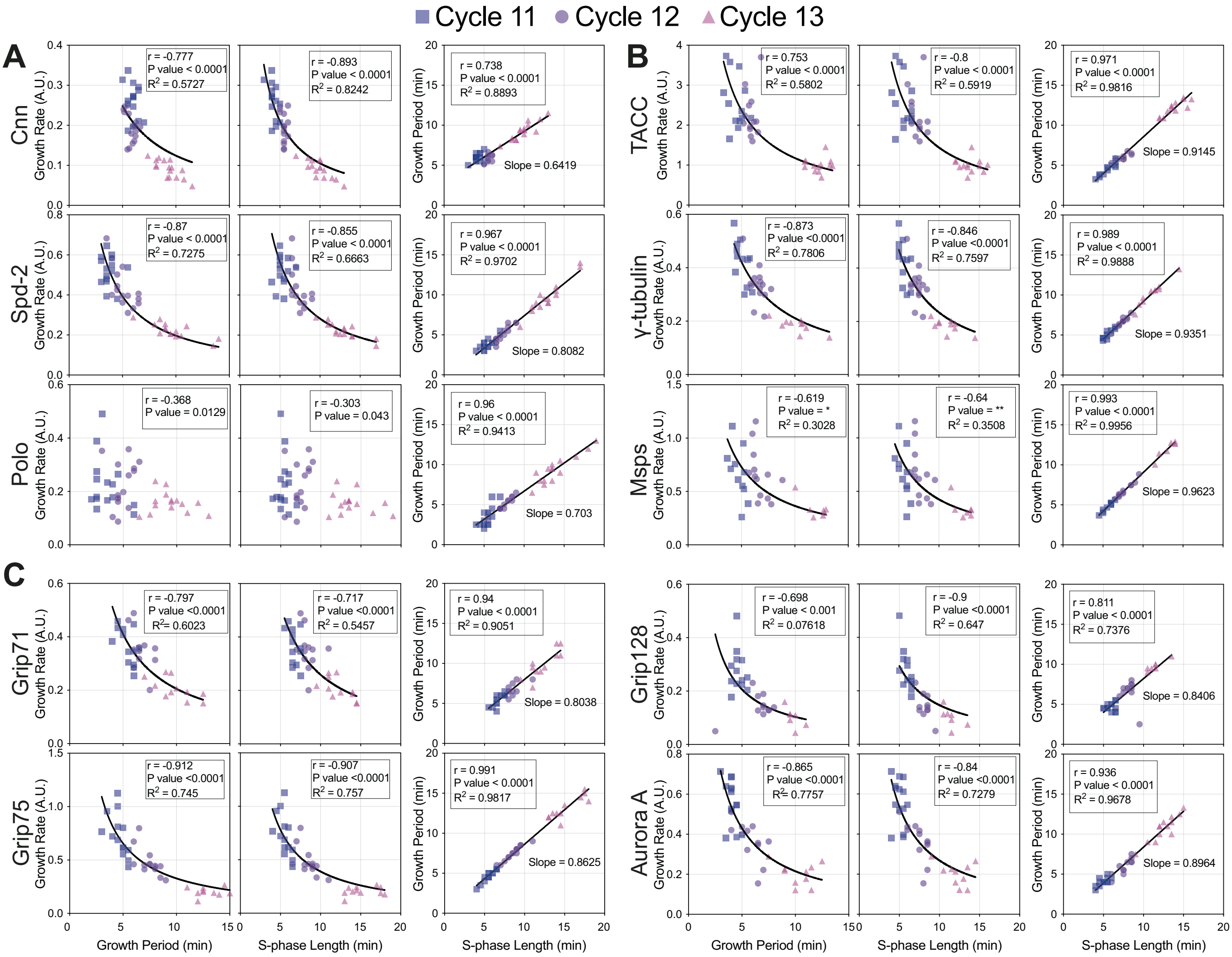


**Figure EV4. Analysis of the strength of correlation between various centrosome growth parameters during NC11, 12 and 13.**

(**A**) Scatter plots show the correlation between the centrosome growth rate and period (left graphs for each protein), growth rate and S-phase length (middle graphs for each protein), and growth period and S-phase length (right graphs for each protein) for the centrosome scaffold proteins. Each data point represents an individual embryo at either NC11 (deep purple squares), NC12 (light purple circles) and NC13 (pink triangles) (calculated from the data shown in Fig. 1A). Lines indicate mathematically regressed best fits for inverse (i.e., *y* is proportional to 1/*x*; left and middle graphs) and linear (i.e., *y* is proportional to *x*; right graphs) correlations. The goodness of fit ($R^2$), strength of correlation (*r*) and the statistical significance (*P* value) are indicated and were calculated in custom Python scripts and GraphPad Prism by either Pearson test (bivariate Gaussian-distributed) or Spearman test (bivariate non-Gaussian-distributed data). Bivariate Gaussian distribution was tested by Henze–Zirkler test. Note that for Polo, the correlation between the centrosome growth rate and either the centrosome growth period or S-phase length did not fit an inverse function well, although the trend was still significant ($P < 0.05$). This suggests that this relationship may be more complicated than for the other proteins, perhaps because Cdk/Cyclins and Polo influence each other's behaviour in multiple ways. (**B**, **C**) Scatter plots show the same as in (**A**) but for the Class I (**B**) of Class II (**C**) client proteins.

 © The Author(s)

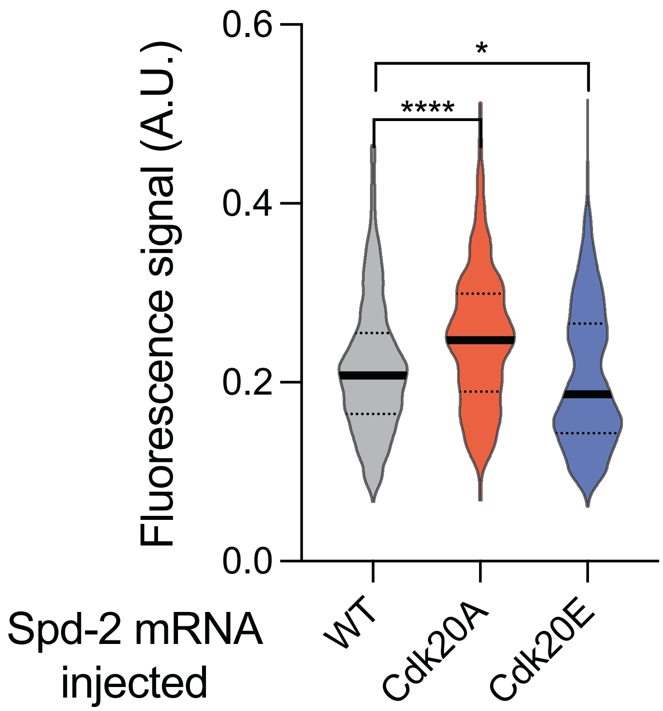


**Figure EV5. Comparison of the centrosomal levels of WT Spd-2-NG, Spd-2-Cdk20A-NG or Spd-2-Cdk20E-NG assayed by mRNA injection.**

Violin plots quantify the centrosomal fluorescence intensity in embryos injected with mRNA encoding either WT Spd-2-NG, Spd-2-Cdk20A-NG or Spd-2-Cdk20E-NG. Horizontal bars indicate the median±quartile. Embryos were injected with mRNA (which is rapidly translated), and the fluorescence intensity of the 50 brightest centrosomes in each embryo were assayed in mid-S-phase (when Spd-2 levels are maximal) ~1 h later. $N = 12–15$ embryos, $n = 600–750$ centrosomes. Statistical significance was computed using a Kruskal–Wallis test, followed by a Dunn's multiple comparisons test (*$P < 0.05$, ****$P < 0.0001$).

© The Author(s)
